# Dysentery as the First Manifestation of Severe COVID-19

**DOI:** 10.7759/cureus.20368

**Published:** 2021-12-12

**Authors:** Alec Seidman-Sorsby, Alberto Moscona-Nissan, Mayte Cruz-Zermeño, Alberto González-Chávez

**Affiliations:** 1 School of Medicine, Universidad Panamericana, Mexico City, MEX; 2 General Surgery, Hospital Español, Mexico City, MEX

**Keywords:** bloody diarrhea, clinical infectious medicine, surgical case report, dysentery, covid 19

## Abstract

The presence of dysentery as the first manifestation of coronavirus disease 2019 (COVID-19) is highly atypical and it may present with concomitant respiratory symptoms or as a single manifestation. Diagnosis is often difficult due to its clinical presentation similar to gastrointestinal diseases, such as infectious diarrhea. We present a case of a 35-year-old male who presented with dysentery as the first manifestation of severe COVID-19.

## Introduction

In December 2019, several cases of atypical pneumonia similar to severe acute respiratory syndrome (SARS) and Middle East respiratory syndrome (MERS) were reported in Wuhan, China. Months later, coronavirus disease 2019 (COVID-19) was known to be caused by a betacoronavirus (severe acute respiratory syndrome coronavirus 2 or SARS-CoV-2), and its genome constituted a single strand of ribonucleic acid (RNA). The infectious agent uses the angiotensin-converting enzyme receptor 2 (ACE-2) to enter cells. The clinical picture of this disease can range from mild symptoms to respiratory failure. Contrary to what was believed, the entire population regardless of age is susceptible to infection. The most common symptoms in affected patients are fever, fatigue, anosmia, and dry cough. As of this date, the gold standard for diagnosis is the polymerase chain reaction (PCR) test to identify the genetic material of the virus [1].

Some patients can have gastrointestinal symptoms, which include anorexia, nausea, vomiting, and diarrhea. As previously mentioned, the damage produced by the virus in the gastrointestinal tract is done through the ACE-2 receptor, which can be found on the mucosa of these organs. The presence of such symptoms can be problematic, due to the potential risk of transmission in patients who are not suspected of a COVID-19 infection, due to their extra-respiratory presentation [2]. As previously mentioned, most patients have respiratory symptoms and fever; however, extra-respiratory manifestations can be present with a lower incidence. Extra-respiratory manifestations can include cardiovascular, neurological, hepatic, renal, cutaneous, hematologic, and gastrointestinal alterations. These presentations may be caused by direct invasion of the virus. Clinicians should be familiar with respiratory and extra-respiratory manifestations to reduce the risk of overlooking patients with COVID-19 infection, taking into consideration that early diagnosis is associated with a better prognosis and lower risk of complications [3].

## Case presentation

A 35-year-old male presented to the emergency department due to a seven-day history of nausea, gastric content vomiting, and soft stools with undigested blood and mucus, having frequent bowel movements (five a day). The patient traveled to Peru three days before symptoms onset and was prescribed nitazoxanide with no improvement. On physical examination, the patient presented with fever (101.3° Fahrenheit) and oxygen saturation of 95% in room air. No abnormalities were found on chest auscultation or tachypnea. The patient had pain on palpation of the right upper quadrant. A single dose of azithromycin was prescribed for traveler's diarrhea. A few hours later, the patient was treated with acetaminophen in the ED for his abdominal pain and fever. In the emergency department, a polymerase chain reaction (PCR) test for SARS-CoV2, stool studies, and a toxin test for *C. difficile *were performed, which were all negative. A complete blood count (CBC) revealed 5,700 white blood cells (WBC) per microliter; 57% of the white blood cells were neutrophils and a complete metabolic panel (CMP) was requested, which revealed a creatinine level of 1.0 mg/dl and normal liver function. Upon admission, intravenous ciprofloxacin was administered; nevertheless, diarrhea and fever persisted. A new CBC was performed, revealing 14,400 WBC per microliter, 16% of band cells, and a 1.68 ng/mL procalcitonin. Additionally, molecular testing of feces was requested, revealing no alterations.

**Figure 1:**
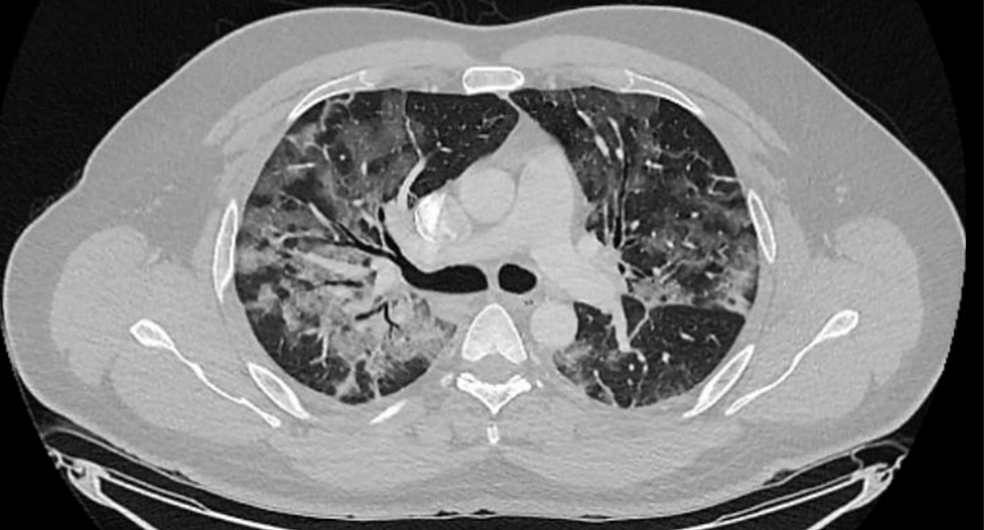
Coronal computed tomography of the chest.

Three days upon admission, fever persisted and the patient referred dyspnea with an oxygen saturation of 85%. Therefore, supplementary oxygen at 3 L/min was administered through a nasal cannula. Despite oxygen therapy, the patient's oxygen saturation fell to 77%, requiring 10 L/min oxygen administration through a mask with a reservoir bag. An arterial gasometry reported a partial pressure of oxygen of 39.6 mm/Hg and a PCR test was positive for SARS-CoV2 infection. A computed tomography (CT) revealed pneumonitis with septal thickening and bilateral consolidations accompanied by enlarged mediastinal lymph nodes (Figure 1). Findings correspond to a COVID-19 Reporting and Data System (CO-RADS) 5 score, highly suggestive of COVID-19. Other inflammatory markers found were procalcitonin of 19.69 ng/mL, C-reactive protein of 11.1 mg/L, and D-dimer of 1,644.52 ng/mL. Therapeutic management included steroids, enoxaparin, and oxygen supplementation; the patient presented a favorable outcome and was discharged 14 days upon admission.

## Discussion

The SARS-CoV-2 virus has caused the global health threat of COVID-19, affecting over 200 million individuals and causing more than 5 million deaths worldwide. The most affected organs are the lungs, due to the presence of the ACE-2 receptor, which is prevalent in type 2 alveolar cells. The virus uses the spike protein to bind to ACE-2 receptors and enter the host cells.

The most common clinical manifestations of SARS-CoV-2 virus infection are respiratory, which include shortness of breath, sputum production, and cough. Patients who are diagnosed with a mild presentation of COVID-19 have predominantly upper airway manifestations as nasal congestion, sore throat, and hyposmia [3]. In the gastrointestinal tract, ACE-2 receptors have been found to be positive in the cytoplasm of gastrointestinal epithelial cells, particularly in the gastric, duodenal, and rectal glandular epithelial cells [4]. The presence of gastrointestinal symptoms in SARS-CoV-2 infection is not infrequent. As demonstrated by Schmulson et al., its prevalence is 3-40% [4]. The most frequent gastrointestinal symptoms in SARS-CoV-2 infection are diarrhea (7.5%), nausea (4.5%), and vomit (1.3%) [3,4]. It is estimated that up to 20% of patients begin with gastrointestinal symptoms, usually diarrhea [5]. When evaluating the presence of gastrointestinal symptoms, if a stool test is requested, positive results can be expected, even after presenting a negative nasopharyngeal swab test. In case of presenting severe diarrhea with complications, an endoscopic intervention may be required [6].

A pathophysiological pathway that has been proposed is that high concentrations of ACE-2 receptors in the intestine and colon may favor the presence of gastrointestinal symptoms [7]. Different case series have reported the presence of SARS-CoV-2 in feces, which has led to the possibility of fecal-oral transmission [8]. Additionally, necrosis of the gastrointestinal mucosa cells due to hypoxia or excessive production of cytokines could be mechanisms that explain gastrointestinal symptoms [8,9].

One of the biggest risk factors in COVID-19, which has a direct correlation with a bad prognosis is obesity, which is defined as a body mass index higher than 30. The following is believed to be associated with a higher concentration of ACE-2 receptors in cells and dysbiosis [10]. The gastrointestinal tract has a microbiota made up of millions of microorganisms, which play a fundamental role in the immune system. Obesity playing a role in the alterations in the microbiota has shown a diminished immune system response, which plays an essential role in the prognosis of COVID-19. The following is associated as well with the presence of gastrointestinal symptoms because as previously mentioned obesity increases the presence of ACE-2 receptors, which leads to a higher susceptibility of having symptoms such as dysentery, anorexia, nausea, and vomit.

## Conclusions

Although the main manifestations of COVID-19 are respiratory, the virus has the ability to affect other organs, including the digestive system. Extra-respiratory manifestations of COVID-19 such as dysentery tend to be overlooked and are considered highly atypical by many. Therefore, understanding the possible clinical manifestations of the disease is fundamental, when making a diagnosis. Nowadays, assessment of gastrointestinal symptoms as dysentery must address COVID-19 as a differential diagnosis. Yet, further research should be conducted to elucidate the pathogenic pathways and mechanisms of COVID-19 gastrointestinal symptoms.
